# Multimorbidity patterns in old adults and their associated multi-layered factors: a cross-sectional study

**DOI:** 10.1186/s12877-021-02292-w

**Published:** 2021-06-19

**Authors:** Jiao Lu, Yuan Wang, Lihong Hou, Zhenxing Zuo, Na Zhang, Anle Wei

**Affiliations:** 1 School of Management, Shanxi Medical University, 56 Xinjian South Road, Taiyuan, 030001 Shanxi Province China; 2 The Second Affiliated Hospital, Shaanxi University of Chinese Medicine, Xianyang, Shaanxi Province China; 3 School of Public Health, Shanxi Medical University, Taiyuan, Shanxi Province China

**Keywords:** Multimorbidity, Patterns, Health ecological model, Multi-layered factors

## Abstract

**Background:**

Influenced by various factors such as socio-demographic characteristics, behavioral lifestyles and socio-cultural environment, the multimorbidity patterns in old adults remain complex. This study aims to identify their characteristics and associated multi-layered factors based on health ecological model.

**Methods:**

In 2019, we surveyed a total of 7480 participants aged 60+ by using a multi-stage random cluster sampling method in Shanxi province, China. Latent class analysis was used to discriminate the multimorbidity patterns in old adults, and hierarchical regression was performed to determine the multi-layered factors associated with their various multimorbidity patterns.

**Results:**

The prevalence of multimorbidity was 34.70% among the old patients with chronic disease. Over half (60.59%) of the patients with multimorbidity had two co-existing chronic diseases. “Degenerative/digestive diseases”, “metabolic diseases” and “cardiovascular diseases” were three specific multimorbidity patterns. Behavioral lifestyles-layered factors had the most explanatory power for the three patterns, whose proportions of explanatory power were 54.00, 43.90 and 48.15% individually. But the contributions of other multi-layered factors were different in different patterns; balanced diet, medication adherence, the size of family and friendship network, and different types of basic medical insurance might have the opposite effect on the three multimorbidity patterns (*p* < 0.05).

**Conclusions:**

In management of old patients with multimorbidity, we should prioritize both the “lifestyle change”-centered systematic management strategy and group-customized intervention programs.

**Supplementary Information:**

The online version contains supplementary material available at 10.1186/s12877-021-02292-w.

## Background

The number of chronic diseases in old patients increases with age, which laid disease burdens to old patients and government [1, 2]. A study revealed the risk of death was 1.73 (95% CI: 1.41; 2.13) and 2.72 (95% CI: 1.81; 4.08) for individuals with 2+ and 3+ co-existing chronic diseases respectively compared with individuals with ≤1 chronic disease [3]. The coexistence of two or more chronic diseases in the same individual is called as “multimorbidity” [4]. This term originated from “comorbidity” [5] but differed from the conception of “comorbidity” that signified a “distinct additional clinical entity” occurring in the setting of an index disease [6, 7]. The conception of “multimorbidity” remains human-centered and focuses on exploring the systematic methods of prevention and intervention of the common risk factors. Frailty can also be used to measure the risk profile of old adults in order to support clinical decisions and design tailored interventions. Frailty refers to a medical syndrome with multiple causes and contributors. It is characterized by diminished strength, endurance, and reduced physiologic function that increases an individual’s vulnerability for developing increased dependency and/or death [8]. Compared with frailty, multimorbidity has a more recognizable measuring standard and implies a more holistic evaluation of the individual’s clinical complexity [9]. To reflect this complexity, it is needed to analyze the differences among co-existing chronic diseases patterns but not the discrepancy in the number of chronic diseases [10, 11]. Besides, the probability of occurrence through specific patterns was higher than that of random disease combinations among patients with multimorbidity.

Recognition of specific patterns will help clinicians predict the possible occurrence of multimorbidity risks among patients and prevent or intervene those risks at group level [12]. However, due to differences in contextual features (ethnic characteristics, living habits, etc.), multimorbidity patterns in different regions and countries also presented differently [13]. Garin et al. analyzed the data from the Collaborative Research on Ageing in Europe project and the World Health Organization’s Study on Global Ageing and Adult Health [14]. They discovered three prevalent multimorbidity patterns around the world, which were “cardio-respiratory” (angina, asthma, and chronic obstructive pulmonary disease), “metabolic” (diabetes, adiposis, and hypertension), and “mental-articular” (arthritis and depression). Sheridan et al. conducted a prospective analysis by using the data from the Survey of Health, Ageing and Retirement in Europe in both 2013 and 2015 [15]. Their results identified 380 unique combinations of chronic disease in old adults with multimorbidity, which proved that hypertension almost existed in each prevalent disease combination. The study of Hernández et al. in Ireland found hypertension and hypercholesterolemia were the most common co-existing diseases by analyzing the chronic diseases combinations from 6101 old adults aged 50+ [12]. Gu et al. identified three multimorbidity patterns (“degenerative disorders”, “digestive/respiratory disorders”, and “cardiovascular/metabolic disorders”) by analyzing the data of old adults aged 60+ in Nanjing, China through exploratory factor analysis [16]. Furthermore, some studies discovered that multimorbidity patterns were also influenced by individual characteristics, such as socio-demographic characteristics, behavioral lifestyles and socio-economic factors excepting for contextual features [17–20].

To accurately determine multimorbidity patterns and their risk factors, some studies introduced theories of cognitive psychology as the factor screening basis, but they rarely involved comprehensive context features. For example, Singer et al. assessed the relationships between multimorbidity and material, psycho-social and behavioural factors based on the theories of social determinants of health (SDoH), but ignored the role of social and cultural contexts [21]. These weaknesses led to the incomplete understanding of the different types of factors associated with multimorbidity, which calls on future researchers to establish effective multimorbidity prevention and intervention strategy. As to this point, this study introduces health ecological model by involving individual-, inter-personal-, community-, organization-, government- and other multi-layered factors associated with individual’s health [22, 23]. The model, which evolved from an ecological model, shows individuals’ health is affected by innate personal characteristics, psychological behavior characteristics, and macro-environmental factors. Health is the result of the interaction of individual characteristics and contextual features [24, 25]. Thus, in this study, we initially tried to identify multimorbidity patterns in old adults, then analyzed the possible multi-layered factors associated with them based on health ecological model.

## Methods

### Study design and data collection

**Fig. 1:**
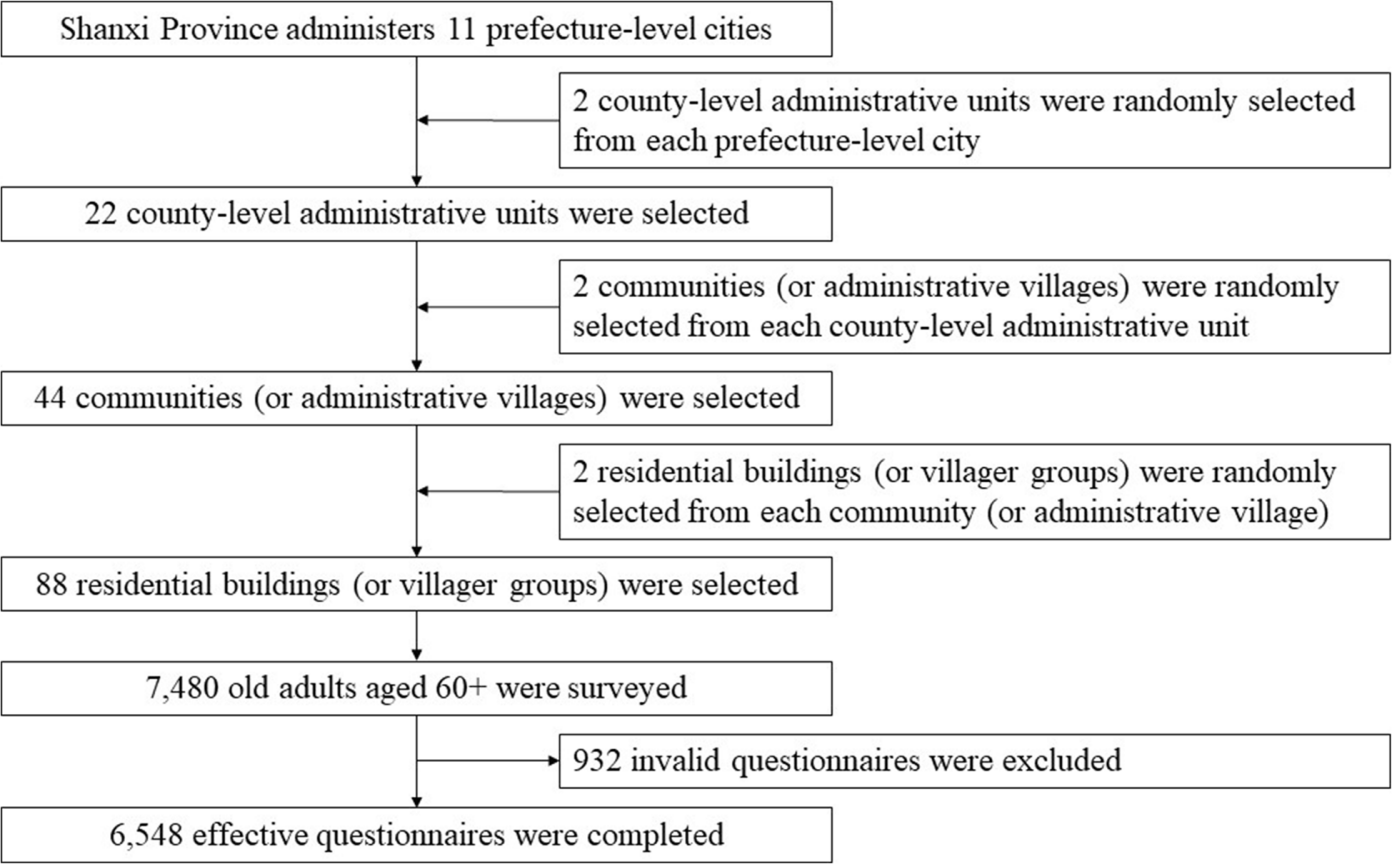
Flowchart of the sampling procedures. All county-level administrative units and communities (or administrative villages) were numbered according to the order listed on the People’s Government of Shanxi Province

We collected data from a cross-sectional questionnaire-based study conducted in Shanxi Province by face-to-face interview. The questionnaire developed for this study is provided as Additional file 1, and some questions refer to the scales developed by Craig et al. [26], Lubben et al. [27], Sherbourne and Stewart [28], and Yu et al. [29]. We used a multi-stage random cluster sampling involving four steps by random-number table. The sampling procedures were shown in Fig. 1. We enrolled all the old adults aged 60+ without cognitive impairment living in the selected residential building/villagers’ groups. Also, in order to ensure the authenticity of the investigation, only old adults who held medical certificates (such as medical records) or current prescriptions could be classified as patients with chronic disease. A total of 7480 urban and rural old adults were surveyed, 932 invalid questionnaires were eliminated, and the effective recovery rate was 87.54%. Before the investigation, all investigators were trained in a centralized manner to ensure that they could exactly understand the standards and procedures of the investigation.

### Variables

#### Outcome variable

To ensure that multimorbidity patterns could be classified effectively, we primarily assessed what kind of chronic diseases should be included in this survey. Some scholars covered both common and uncommon chronic diseases in their survey to guarantee the completeness of investigation, but researchers found that this just increased the complexity of the evaluation but decreased the accuracy of the results [30]. By default, at least 12 kinds of chronic diseases could meet the basic requirement of the investigation on quantity, and they should be determined on their regional prevalence [31]. Therefore, we defined 17 chronic diseases in this study, according to the prevalence of chronic diseases of health statistics yearbook in China (2019) [32], the results of the investigation about the prevalence of chronic diseases in Shanxi Province [33], and the suggestions of clinicians. They were adiposis, hypertension, diabetes, coronary heart disease, stroke, arrhythmia, atherosclerosis, bronchial asthma, chronic obstructive pneumonia diseases, sciatica, arthritis, thyroid diseases, osteoporosis, hearing loss, eye diseases, mental diseases, digestive system diseases and others (the summary of all uncommon chronic diseases). Next, we chose the old adults with two or more co-existing diseases to perform an exploratory latent class analysis (LCA), then clustered them into different latent class groups based on the definition of 17 chronic diseases. The result would be used as the observation category variables for subsequent analysis.

#### Independent variable

**Fig. 2:**
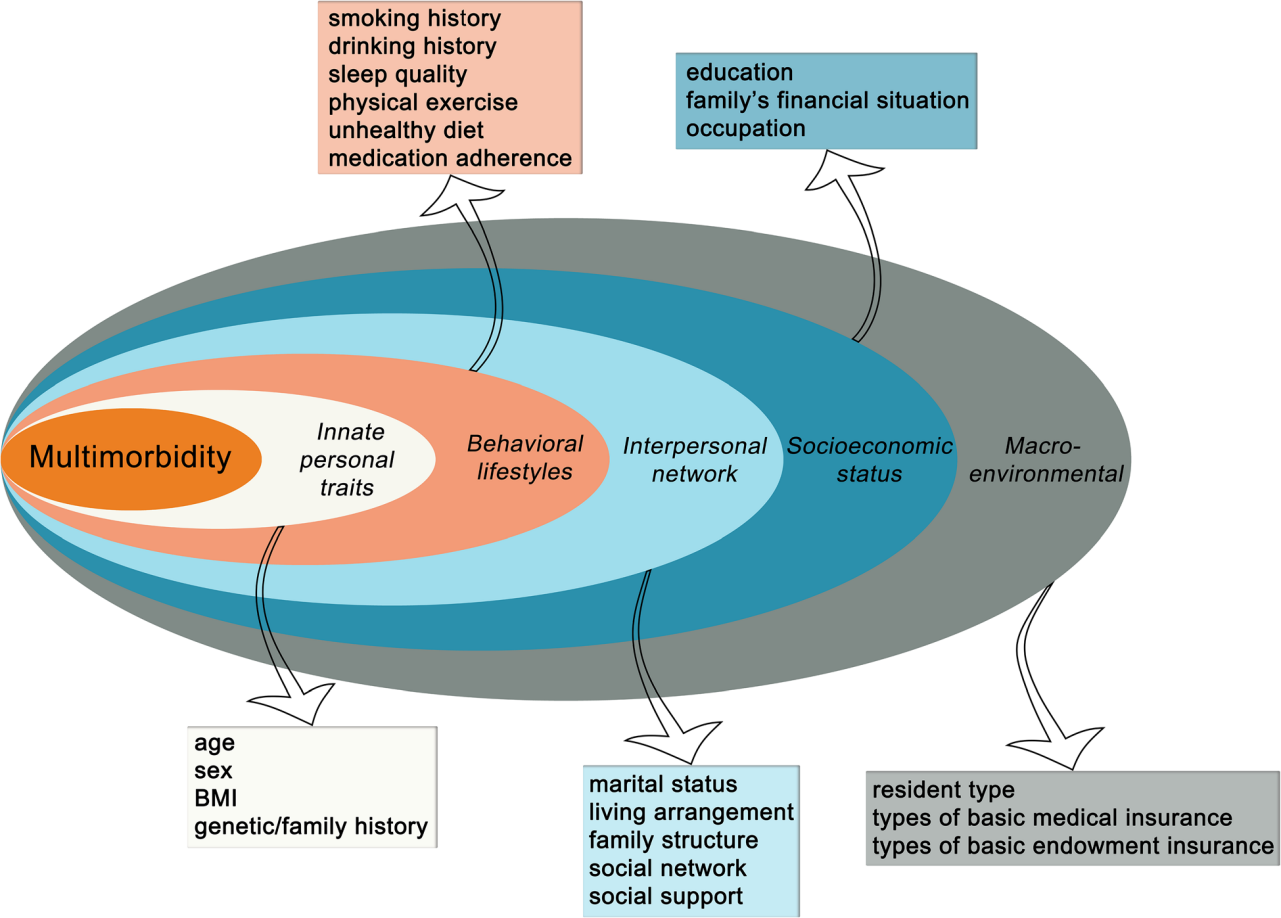
Health ecological model

By the comprehensive and systemic literature review of multimorbidity, the factors which influenced the old adults with multimorbidity were integrated in all multi-layered of health ecological model in this study (Fig. 2). The variable assignments and measurement scales are shown in Table 1.

**Table 1:** Assignments and measurement scales of independent variables (*N* = 1860)

Layer factors	Independent variables	Assignments	N (%)	Mean ± SD
I Innate personal trait	Age	age (years)		70.79±6.954
	Sex	female, male	973(52.3) / 887(47.7)	
	BMI	height/weight2 (kg/m)		23.05±3.677
	Genetic/family history	no, yes	1676(90.1) / 184(9.9)	
II Behavioral lifestyle	Smoking history	current, quit, never	297(16.0) / 232(12.5) / 1331(71.6)	
	Drinking history	current, quit, never	308(16.6) / 167(9.0) / 1385(74.5)	
	Sleep quality	very good, good, poor, very poor	487(26.2) / 981(52.7) / 338(18.2) / 54(2.9)	
	Physical exercise	low level, moderate level, high level	1792(96.3) / 68(3.7)	
	Balanced diet	no, yes	930(50.0) / 930(50.0)	
	Light diet	no, yes	702(37.7) / 1158(62.3)	
	Regular meals	no, yes	477(25.6) / 1383(74.4)	
	Consumption of coarse cereals (g)	0-100, 101-200, 201-300, 301-400, 401-	63(3.4) / 224(12.0) / 415(22.3) / 731(39.3) / 427(23.0)	
	Consumption of fruits and vegetables (g)	0-100, 101-200, 201-300, 301-400, 401-	39(2.1) / 154(8.3) / 243(13.1) / 622(33.4) / 802(43.1)	
	Medication adherence	0-8		4.30±2.838
III Interpersonal network	Marital status	married, unmarried, divorced, widowed	24(1.3) / 1413(76.0) / 17(0.9) / 406(21.8)	
	Living arrangement	live alone, live with others	268(14.4) / 1592(85.6)	
	Family structure	empty-nest, non-empty-nest	892(48.0) / 968(52.0)	
	Family network	LSNS-6 Family subscale		12.02±2.893
	Friendship network	LSNS-6 Friends subscale		8.70±4.208
	Social support	MOS-SSS-C		71.79±14.408
IV Socio-economic status	Education	primary school and below, junior school, high school and above	905(48.7) / 509(27.4) / 446(24.0)	
	Per capita monthly family income (¥)	≤1000, 1001-3000, 3001-5000, >5000	712(38.3) / 614(33.0) / 422(22.7) / 112(6.0)	
	Pre-retirement occupation	public functionary, farmer, self-employed, unemployed, others	585(31.5) / 775(41.7) / 167(9.0) / 85(4.6) / 248(13.3)	
V Macro-environmental	Resident type	rural, urban	982(52.8) / 878(47.2)	
	Types of basic medical insurance	urban employee basic medical insurance, urban and rural resident medical insurance, others	558(30.0) / 1138(61.2) / 164(8.8)	
	Types of basic endowment insurance	urban employee basic endowment insurance, urban and rural resident endowment insurance, others	475(25.5) / 1326(71.3) / 59(3.2)	

### Statistical analyses

The data was input and checked by EpiData3.1, and the diseases status of old adults with multimorbidity was described by the number of diseases (N) and percentage (%). Meanwhile, LCA was used to identify the multimorbidity patterns, and hierarchical logistic regression analysis was applied to determine the multi-layered factors associated with various multimorbidity patterns for old adults. Specifically, multi-layered predictors were tested step-by-step for their prediction of the outcome variables, and five models were established in each multimorbidity pattern respectively: model I incorporated the first-layer factor (innate personal traits); the second-layer factor (behavioral lifestyles) was added in model II on the basis of the model I; similarly, model III, model IV and V introduced the third-layer factor (interpersonal networks), the forth-layer factor (socio-economic status) and the fifth-layer factor (macro-environmental) individually on the basis of model II, model III and model IV. We applied Mplus 7.4 to perform LCA and Stata15.1 to perform hierarchical logistic regression analysis, and statistically significant level was set at 0.05.

## Results

### Descriptive results

In total, 1188 (18.14%) old adults did not suffer from any chronic disease, and 34.70% of patients with chronic disease suffered from multimorbidity. The mean age of 1860 old adults with multimorbidity was (70.79 ± 6.954) year-old. The majority of multimorbidity old patients suffered from two co-existing chronic diseases (60.59%).

**Fig. 3:**
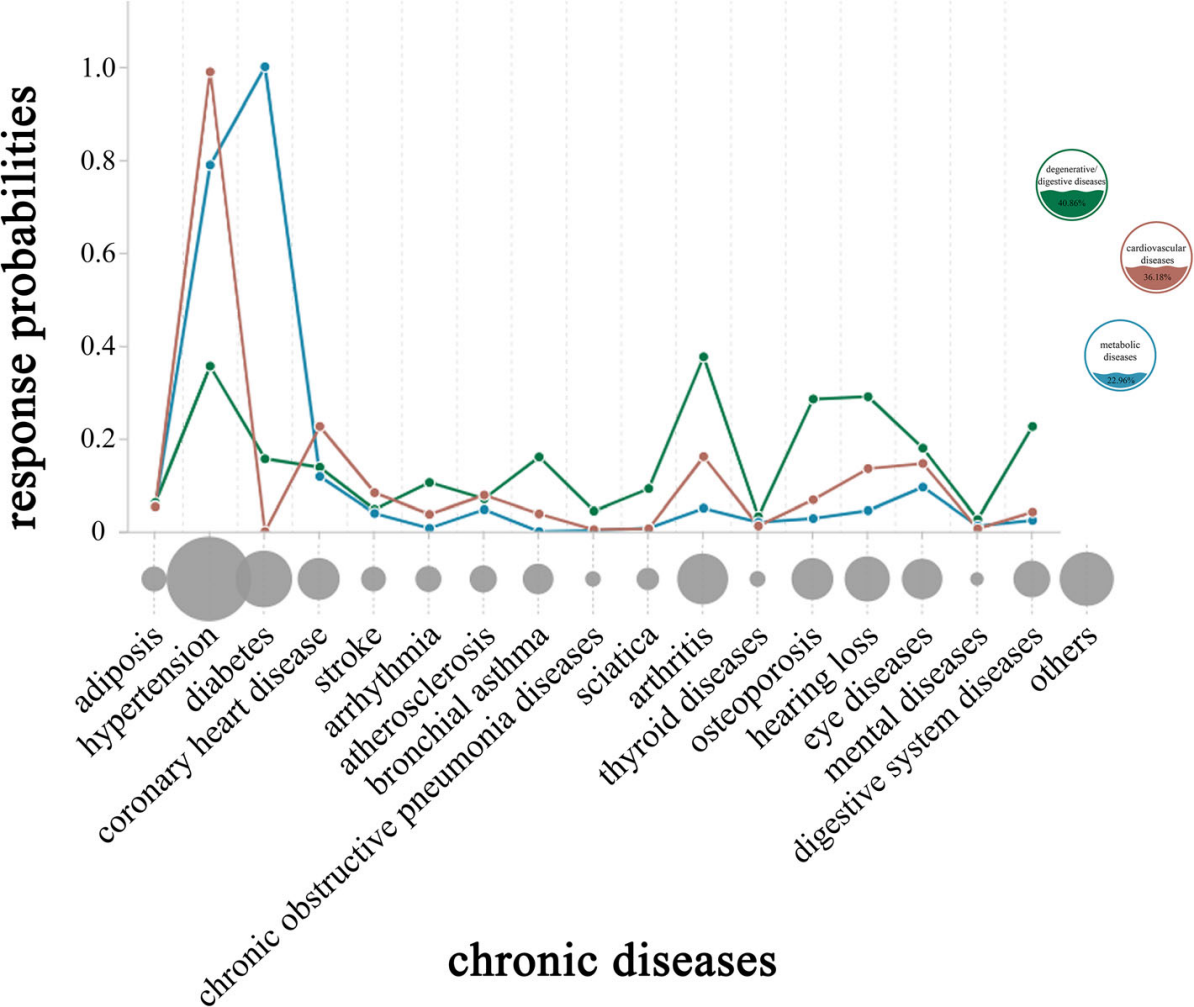
A combined figure. The gray bubbles on the horizontal axis represent the prevalence of each disease in patients with multimorbidity, and the line graph on the vertical axis represents the observation items of each potential class in the results of the LCA, that is, the response probability of 17 chronic diseases. The bubble size in the legend indicates the proportion of the three multimorbidity patterns. The green bubble and line graph depict the data of “degenerative/digestive diseases”, the brown bubble and line graph describe the data of “cardiovascular diseases”, and the blue bubble and line graph delineate the “metabolic diseases”

At the same time, 6 cluster models were extracted in this study by using LCA. From Table 2, by comparing the Bayesian Information Criterion (BIC), the *p*-value of the Bootstrap Likelihood Ratio Test (BLRT) and the interpretability of each class model, the 3-class model was chosen as the optimal one [34]. Moreover, the average probability (column) of the old patients (rows) in multimorbidity of each class ranged from 80.3 to 91.4% in the 3-class model (Table 3), and it also showed the credibility of the 3-class model. The response probability graph of the 3-class (Fig. 3) presents multimorbidity old patients in the pattern-I had higher probability of suffering from arthritis, hearing loss, osteoporosis, and digestive system disease, and lower probability of suffering from hypertension, which could be named as “degenerative/digestive diseases”; those in the pattern-II had nearly 100% probability of suffering from diabetes and were also vulnerable to get hypertension, which could be called as “metabolic disease”; the prevalence of hypertension in the pattern-III was close to 100%, and even there was about 20% probability of coronary heart disease, which could be named as “cardiovascular disease”. The proportions of these three multimorbidity patterns were 40.86, 22.96 and 36.18% respectively.

**Table 2:** Model-fit statistics comparison for latent class analysis

Model	k	AIC	BIC	aBIC	Entropy	LMR	BLRT
1 Class	17	21,073.821	21,167.803	21,113.794	–	–	–
2 Classes	35	20,627.163	20,820.654	20,709.460	0.485	<.0001	<.0001
3 Classes	53	20,525.961	20,818.962	20,650.582	0.729	<.0001	<.0001
4 Classes	71	20,468.246	20,860.758	20,635.192	0.793	0.4490	<.0001
5 Classes	89	20,413.296	20,905.317	20,622.566	0.857	0.0009	<.0001
6 Classes	107	20,382.435	20,973.967	20,634.030	0.868	0.9101	<.0001

*k* Number of Free Parameters, *AIC* Akaike Information Criterion, *BIC* Bayesian Information Criterion, *aBIC* Adjusted Bayesian Information Criterion, *LMR* Lo-Mendell-Rubin Likelihood Ratio Test, *BLRT* Bootstrap Likelihood Ratio Test

**Table 3:** The average probability (column) of the old patients (rows) in multimorbidity of each class

	Class 1 (%)	Class 2 (%)	Class 3 (%)
Class 1	91.4	2.6	6.0
Class 2	8.6	91.4	0.0
Class 3	19.7	0.0	80.3

### Sensitivity analysis

Provided that patients with uncommon chronic diseases were also recruited as our respondents, the accuracy of the multimorbidity patterns may be affected. Thus, to assess the reliability of those patterns, we re-selected samples by excluding those individuals belonging to the ‘others’ category to conduct a sensitivity analysis. We used the LCA to analysis the data of 1581 multimorbidity patients suffering from these 17 common chronic diseases. The results also identified three specific multimorbidity patterns: “degenerative/digestive diseases”, “metabolic diseases” and “cardiovascular diseases”; and that was similar to the original ones. More detailed information is included in Additional file 2.

### Hierarchical regression analysis results

The results showed that the degree of interpretation of the model could continuously rise with the addition of multi-layered factors; the behavioral lifestyles-layered factors explained the most for the three patterns, and their proportions of explanatory power were 54.00, 43.90 and 48.15% individually. But specific factors in multi-layered associated with three multimorbidity patterns were significantly different (Table 4).

**Table 4:** Hierarchical regression analysis of the factors associated with multimorbidity patterns

Layer factors	Independent variables	OR		
		Pattern 1	Pattern 2	Pattern 3
Innate personal trait I	Age	-	-	1.02**
	Genetic/family history			
	no	Reference		
	yes	-	1.67***	-
II Behavioral lifestyle	Sleep quality			
	very good	Reference		
	good	-	-	-
	poor	-	-	-
	very poor	1.97**	-	-
	Physical exercise			
	low level	-	-	-
	moderate level	Reference		
	high level	-	-	0.15**
	Balanced diet			
	no	1.40***	0.70***	-
	yes	Reference		
	Light diet			
	no	-	-	1.28**
	yes	Reference		
	Medication adherence	0.92***	1.05**	1.05***
III Interpersonal network	Family network	1.07***	0.94***	-
	Friendship network	0.97**	1.03**	-
	Social support	0.99**	-	-
IV Socio-economic status	Education			
	primary school and below	Reference		
	junior school	-	0.69**	-
	high school and above	-	-	-
	Per capita monthly family income (¥)			
	0-1000	Reference		
	1001-3000	-	-	-
	3001-5000	1.56**	-	-
	5001-	-	-	-
V Macro-environmental	Types of basic medical insurance			
	urban employee basic medical insurance	0.49***	2.58***	-
	urban and rural resident medical insurance	0.66**	1.88**	-
	others	Reference		

*Note*. Only significant variables are shown here. Standard errors are in parenthesis *** *p*<0.01, ** *p*<0.05

Regarding the “degenerative/digestive diseases” pattern, the factors in innate personal traits layer failed to significantly predict the “degenerative/digestive diseases” but other layers could predict it. The probability of getting “degenerative/digestive diseases” may rise in patients with very poor sleep quality, an imbalanced diet, great size of the family network and upper-middle-income (Ұ3001-5000). Factors such as good medication adherence, great scale of friendship network, high social support and participation in urban employee basic medical insurance (UEBMI) and urban and rural resident medical insurance (URRMI) might reduce the probability of this disease pattern.

With regard to the “metabolic diseases” pattern, five multi-layered factors were all predicted significantly. The genetic/family history, good medication adherence, great size of friendship network and participation in the UEBMI and URRMI, were risk factors for “metabolic diseases” pattern. However, an imbalanced diet, the extension of the family network’s size, and junior school education were protective factors for this pattern.

As to the “cardiovascular diseases” pattern, only innate personal traits-layered factor and behavioral lifestyles-layered factor significantly predicted the “cardiovascular diseases”. Aging, heavy oil and high-salt diet and good medication adherence, might rise the probability of patients suffering from “cardiovascular diseases”; while high-intensity physical exercise might significantly reduce it.

## Discussion

We identified “degenerative/digestive diseases”, “metabolic diseases” and “cardiovascular diseases” as the three specific patterns of multimorbidity among Chinese old patients with chronic disease in this study. The pattern of degenerative/digestive diseases is comprised of similar proportions of arthritis, hypertension, hearing loss, osteoporosis, digestive system diseases, eye diseases, and its prevalence was 40.86%. Degenerative diseases as a separate pattern were observed in Chinese old populations but not in other countries or regions [16, 35–40]. Gu et al. used data from the community-dwelling old persons in Nanjing, China and discovered hearing disorder, cataract, joint disease and cancer were included in the degenerative diseases group [16, 38], but cancer was not included in our and other studies [35–37, 39]. Meanwhile, the digestive system diseases and arthritis diseases were often clustered (stomach or other digestive disease, arthritis) among older Chinese [40, 41]. Moreover, the patterns of cardiovascular diseases and metabolic diseases were observed in almost all studies focusing on older Chinese [16, 38, 39, 41, 42]. In this study, the pattern of metabolic diseases was characterized by having 100% probability to suffer from diabetes and 78.9% probability of being ill with hypertension, and its prevalence was 22.96%. While more than 1 in 5 persons in the pattern of cardiovascular diseases had coronary heart-hypertension combination, and the prevalence of this pattern was 36.18%. The study of She et al. found that the pattern of cardiovascular disorders (arrhythmia, ischemic heart disease, and heart failure) and the pattern of metabolic disorders (hypertension, obesity, diabetes, and dyslipidemia) were identified by the 1497 rural community older from the Confucius Hometown Aging Project (2014-2016) in Qufu, Shandong, China [39]. Hypertension, diabetes, coronary heart disease were also the major diseases of the cardiovascular and metabolic diseases patterns in other studies [16, 38, 41]. However, mental illness did not appear in disease combination in this study. This finding was inconsistent with some previous studies. Garin et al. observed a “Mental-articular” pattern in China, Ghana and India, which included arthritis and depression and “Mental-articular” pattern in Spain, which included arthritis, depression and anxiety [14, 43]. This may be limited by the sample size. The small sample size of mental illnesses leads to the low joint probability and conditional probability of mental illnesses and other chronic diseases [34], resulting in a low probability of mental illnesses appearing in the multimorbidity patterns in our study.

Remarkable heterogeneity in the number, types, and assessment approaches of chronic conditions [39], as well as the aging characteristics of different study samples [16], has led to some differences in the combination of patterns across studies, but specific common multimorbidity patterns have been identified, such as metabolic syndromes, degenerative and cardiovascular diseases.

What’s more interesting, some specific multi-layered factors were protective factors for one pattern, but risk factors for another. An imbalanced diet might cause gastrointestinal mucosal damage and dysfunction which might increase the incidence of “degenerative/digestive diseases”. While, “metabolic diseases” always require an accurate and strict restriction on the intake of foods rich in fats, protein, starch and sugar, so a reasonable diet might increase the probability of this disease. A great scale and close-contact of friendship network might enhance the patients’ mutual assistance and information exchange in chronic disease management to reduce the incidence of such “degenerative/digestive diseases” pattern [21, 44, 45]. But individuals may be opposed to disease management because of the negative emotions brought about by excessive constraints from family members. Some unreasonable lifestyle habits might be aggrandized with the expansion of the friendship network which increase the incidence of “metabolic diseases” pattern. It is worth mentioning that good medication adherence might no longer be beneficial for all disease patterns. In the early stages of disease, rational administration might prevent functional injuries and reduce the incidence of “degenerative/digestive diseases”. However, long-term use of certain drugs would lead to the “metabolic diseases” pattern [46]. For example, long-term use of atypical antipsychotic drugs (AAP) might increase the risk of weight gain, blood sugar and blood lipids rise and insulin resistance, which would lead to adiposis, diabetes, high blood pressure, etc. [47]. In addition, medication adherence might also increase the risk of “cardiovascular diseases” in multimorbidity patients. Ferdinandy et al. discovered that some comorbidities and their medications might have potential cardiotoxity of a drug [48]. Medication is the preferred way to control disease progression, but to avoid adverse drug interactions that further complicate the disease, we should pay attention to the adverse effects of long-term medication on certain multimorbidity patterns. In addition, except for the factors above, genetic/family history might increase the risk of “metabolic diseases” [49, 50]. Aging and heavy oil and high-salt diet were the risk factors for “cardiovascular disease” [51, 52], while high-intensity physical exercise was a protective factor [53].

There were four limitations in this study. First, cross-sectional data in this study failed to consider the time effect on multimorbidity, and the causality inference could not be estimated. Meanwhile, although our investigators were strictly trained and had medical professional backgrounds, self-reported variables in our study also may be misestimated inevitably. A large-scale longitudinal study should be designed to validate these possible causal hypotheses in future more accurately. Second, the data in this study were investigated from a single geographical region, which might be difficult to verify the effect of macro-environmental factors effectively. Thus, the conclusion of this study should be applicable to regions with the same political and cultural environment. Future studies should expand the research area to the whole China or even more countries. Third, the proportion of old adults with multimorbidity may be underestimated because respondents who have suffered from chronic illnesses but not diagnosed by doctors may be excluded in our study. Moreover, although most Chinese older people choose home-based care, the chronically ill older people who lived in nursing homes or hospitals were not interviewed. Fourth, the variables involved in this study might not be comprehensive enough to cover all associated factors, although these variables were selected based on health ecological model and systemic literature review. Some other variables such as home and community living environment should be further analyzed in future studies.

## Conclusions

In this study, we identified three specific multimorbidity patterns, “degenerative/digestive diseases”, “metabolic diseases” and “cardiovascular diseases”, among old Chinese patients. The behavioral lifestyles-layered factors mostly explained three multimorbidity patterns, but specific factors of different layers in the three multimorbidity patterns were significantly different, and some factors might even have opposite effects on different multimorbidity patterns. Therefore, a multi-level “individual-community-government” systematic management strategy should be established to manage old patients’ multimorbidity, considering both the “lifestyle change”-centered systematic management strategy and group-customized intervention program for different multimorbidity patterns. From the perspective of individuals, the self-management ability of old patients with chronic diseases should be improved in continuous visualized education and decision-making to provide more support in health-promotion and disease-prevention behaviors. From the perspective of communities, the multimorbidity care service delivery system should be strengthened by firstly building a knowledge-sharing network to facilitate the peer effect of patient group with multimorbidity, and by secondly establishing classified and graded archives and remote dynamic risk monitoring and early warning system for multimorbidity patients to help formulate group-customized intervention programs. From the perspective of the government, a unified and coordinated two-way referral tactics should be developed to improve the management of multimorbidity, and give full play to the catch-all and mutual-aid roles of basic medical insurance.

## Supplementary Information

**Additional file 1.** Questionnaire for old adults

**Additional file 2.** Sensitivity analysis

## Data Availability

The datasets generated and analyzed during the current study are available from the corresponding author on reasonable request.

## Abbreviations

LCA: Latent class analysis
BIC: Bayesian Information Criterion
BLRT: Bootstrap Likelihood Ratio Test
AAP: Atypical antipsychotic drugs
SDoH: Theories of social determinants of health
UEBMI: Urban employee basic medical insurance
URRMI: Urban and rural resident medical insurance
