# Supplementary material for: Multimorbidity patterns in old adults and their associated multi-layered factors: a cross-sectional study
Source: BMC Geriatr. 2021 Jun 19;21:372. doi: 10.1186/s12877-021-02292-w (PMC8214251; doi:10.1186/s12877-021-02292-w)
Supplement: Supplementary file 1 — Additional file 1. Questionnaire for old adults [file 12877_2021_2292_MOESM1_ESM.docx]

**Additional File 1: Questionnaire for old adults**

**Demographic Backgrounds**

**1. Age (years):**

**2. Sex:**

① female

② male

**3. Height (cm):**

**4. Weight (kg):**

**5. Marital status:**

① married

② unmarried

③ divorced

④ widowed

**6. Living arrangement:**

① live alone

② live with others

**7. Family structure:**

① empty-nest

② non-empty-nest

**8. The highest grade of school or year of college you completed:**

① primary school and below

② junior school

③ high school and above

**9. Per capita monthly family income (¥):**

① ≤1000

② 1001-3000

③ 3001-5000

④ >5000

**10. Pre-retirement occupation:**

① public functionary

② farmer

③ self-employed

④ unemployed

⑤ others

**11. Resident type:**

① rural

② urban

**12. Types of basic medical insurance:**

① urban employee basic medical insurance

② urban and rural resident medical insurance

③ others

**13. Types of basic endowment insurance:**

① urban employee basic endowment insurance

② urban and rural resident endowment insurance

③ others

**Health conditions and Medication adherence**

**1. Have you been diagnosed with chronic diseases listed below by a doctor?**

| **Chronic diseases** | **Yes** | **No** |
| --- | --- | --- |
| adiposis |  |  |
| hypertension |  |  |
| diabetes |  |  |
| coronary heart disease |  |  |
| stroke |  |  |
| arrhythmia |  |  |
| atherosclerosis |  |  |
| bronchial asthma |  |  |
| chronic obstructive pneumonia diseases |  |  |
| sciatica |  |  |
| arthritis |  |  |
| thyroid diseases |  |  |
| osteoporosis |  |  |
| hearing loss |  |  |
| eye diseases |  |  |
| mental diseases |  |  |
| digestive system diseases |  |  |
| [Please listed other chronic diseases you have here]  _________________________________________ | | |

**2. Does your family have the above chronic disease?**

① yes

② no

**3. Medication adherence**

| **Items** | **Yes** | **No** |
| --- | --- | --- |
| Do you sometimes forget to take your medicine? |  |  |
| Over the past seven days, do you sometimes forget to take your medicine? |  |  |
| Have you ever decided to stop taking your medicine on you own when you feel that taking medicine is making you worse? |  |  |
| Do you sometimes forget to take your medicine with you when you are out? |  |  |
| Have you taken your medicine regularly in the past seven days? |  |  |
| Have you ever decided to stop taking your medicine on you own when you feel that taking medicine is making you better? |  |  |
| Do you sometimes forget to stick to your treatment plan? |  |  |
| Do you sometimes forget to take all your medicine? |  |  |

**Behavioral lifestyles**

**1. Have you ever smoked cigarettes?**

① still have

② quit

③ never smoked

**2. Have you ever drink alcoholic beverages?**

① still have

② quit

③ never had a drink

**3. How was your sleep quality?**

① very good

② good

③ poor

④ very poor

**4. Physical exercise^[[1]](#endnote-2)^**

|  | **During a usual week, did you do … for at least 10 minutes continuously?** | **During a usual week, on how many days did you do … for at least 10 minutes?** | **How much time did you usually spend doing … on one of those days?** |
| --- | --- | --- | --- |
| Think about the time you spend walking in a usual week. This includes at work and at home, walking to travel from place to place, and any other walking that you might do solely for recreation, sport, exercise, or leisure. | ① yes  ② no | days | ① ＜ 30 minutes  ② 30 minutes ≤…＜ 2 hours  ③ 2 hours ≤…＜ 4 hours  ④ ≥ 4 hours |
| Think about activities which take moderate physical effort that you do in a usual week. Moderate physical activities make you breathe somewhat harder than normal and may include carrying light loads, bicycling at a regular pace, or mopping the floor. Again, think about only those physical activities that you did for at least 10 minutes at a time. | ① yes  ② no | days | ① ＜ 30 minutes  ② 30 minutes ≤…＜ 2 hours  ③ 2 hours ≤…＜ 4 hours  ④ ≥ 4 hours |
| Think about all the vigorous activities requiring hard/high-intensity physical effort that you do in a usual week. Vigorous activities make you breathe much harder than normal and may include heavy lifting, digging, plowing, aerobics, fast bicycling, and cycling with a heavy load. Think only about those physical activities that you did for at least 10 minutes at a time. | ① yes  ② no | days | ① ＜ 30 minutes  ② 30 minutes ≤…＜ 2 hours  ③ 2 hours ≤…＜ 4 hours  ④ ≥ 4 hours |

**5. Do you keep balanced diet?**

① yes

② no

**6. Do you keep light diet?**

① yes

② no

**7. Do you have regular meals?**

① yes

② no

**8. Daily consumption of coarse cereals (g):**

① 0-100

② 101-200

③ 201-300

④ 301-400

⑤ 401-

**9. Daily consumption of fruits and vegetables (g):**

① 0-100

② 101-200

③ 201-300

④ 301-400

⑤ 401-

**Interpersonal network**

**1. LSNS-6^[[2]](#endnote-3)^**

| **Subscales** | **Items** | **0** | **1** | **2** | **3-4** | **5-8** | **≥9** |
| --- | --- | --- | --- | --- | --- | --- | --- |
| Family | How many relatives do you see or hear from at least once a month? |  |  |  |  |  |  |
|  | How many relatives do you feel close to such that you could call on them for help? |  |  |  |  |  |  |
|  | How many relatives do you feel at ease with that you can talk about private matters? |  |  |  |  |  |  |
| Friends | How many friends do you see or hear from at least once a month? |  |  |  |  |  |  |
|  | How many friends do you feel close to such that you could call on them for help? |  |  |  |  |  |  |
|  | How many friends do you feel at ease with that you can talk about private matters? |  |  |  |  |  |  |

**2. MOS Social Support Survey^[[3]](#endnote-4)^ ^[[4]](#endnote-5)^**

**① About how many close friends and close relatives do you have (people you feel at ease with and can talk to about what is on your mind)?**

**Write in number of close friends and close relatives:**

**② People sometimes look to others for companionship, assistance, or other types of support. How often is each of the following kinds of support available to you if you need it?**

| **Items** | **None**  **of the time** | **A little of the time** | **Some of the time** | **Most**  **of the time** | **All**  **of the time** |
| --- | --- | --- | --- | --- | --- |
| Someone to help you if you were confined to bed |  |  |  |  |  |
| Someone you can count on to listen to you when you need to talk |  |  |  |  |  |
| Someone to give you good advice about a crisis |  |  |  |  |  |
| Someone to take you to the doctor if you needed it |  |  |  |  |  |
| Someone who shows you love and affection |  |  |  |  |  |
| Someone to have a good time with |  |  |  |  |  |
| Someone to give you information to help you understand a situation |  |  |  |  |  |
| Someone to confide in or talk to about yourself or your problems |  |  |  |  |  |
| Someone who hugs you |  |  |  |  |  |
| Someone to get together with for relaxation |  |  |  |  |  |
| Someone to prepare your meals if you were unable to do it yourself |  |  |  |  |  |
| Someone whose advice you really want |  |  |  |  |  |
| Someone to do things with to help you get your mind off things |  |  |  |  |  |
| Someone to help with daily chores if you were sick |  |  |  |  |  |
| Someone to share your most private worries and fears with |  |  |  |  |  |
| Someone to turn to for suggestions about how to deal with a personal problem |  |  |  |  |  |
| Someone to do something enjoyable with |  |  |  |  |  |
| Someone who understands your problems |  |  |  |  |  |
| Someone to love and make you feel wanted |  |  |  |  |  |

**References to individual questions:**

1. Craig CL, Marshall AL, Sjöström M, Bauman AE, Booth ML, Ainsworth BE, et al. International Physical Activity Questionnaire: 12-Country Reliability and Validity. Med Sci Sports Exerc. 2003;35(8): 1381-1395. [↑](#endnote-ref-2)
2. Lubben J, Blozik E, Gillmann G, Iliffe S, von Renteln Kruse W, Beck JC, et al. Performance of an abbreviated version of the Lubben Social Network Scale among three European community-dwelling older adult populations. Gerontologist. 2006;46(4):503-513. [↑](#endnote-ref-3)
3. Sherbourne CD, Stewart AL. The MOS social support survey. Soc Sci Med. 1991;32(6):705-714. [↑](#endnote-ref-4)
4. Yu DS, Lee DT, Woo J. Psychometric testing of the Chinese version of the medical outcomes study social support survey (MOS-SSS-C). Res Nurs Health. 2004;27(2):135-143. [↑](#endnote-ref-5)
