# Supplementary material for: Multimorbidity patterns in old adults and their associated multi-layered factors: a cross-sectional study
Source: BMC Geriatr. 2021 Jun 19;21:372. doi: 10.1186/s12877-021-02292-w (PMC8214251; doi:10.1186/s12877-021-02292-w)
Supplement: Supplementary file 2 — Additional file 2. Sensitivity analysis [file 12877_2021_2292_MOESM2_ESM.docx]

**Additional File 2: Sensitivity analysis**

**Table S1** Sensitivity analysis in model-fit statistics comparison for latent class analysis

| Model | k | AIC | BIC | aBIC | Entropy | LMR | BLRT |
| --- | --- | --- | --- | --- | --- | --- | --- |
| 1 Class | 17 | 19061.110 | 19152.329 | 19098.323 |  | - | - |
| 2 Classes | 35 | 18605.738 | 18793.542 | 18682.354 | 0.514 | <.0001 | <.0001 |
| 3 Classes | 53 | 18435.756 | 18720.144 | 18551.774 | 0.754 | <.0001 | <.0001 |
| 4 Classes | 71 | 18367.993 | 18748.966 | 18523.414 | 0.801 | 0.1648 | <.0001 |
| 5 Classes | 89 | 18320.249 | 18797.806 | 18515.072 | 0.806 | 0.0003 | <.0001 |
| 6 Classes | 107 | 18273.411 | 18847.553 | 18507.636 | 0.833 | 0.0005 | <.0001 |

*Note*. k = Number of Free Parameters; AIC = Akaike Information Criterion; BIC = Bayesian Information Criterion; aBIC = Adjusted Bayesian Information Criterion; LMR = Lo-Mendell-Rubin Likelihood Ratio Test; BLRT = Bootstrap Likelihood Ratio Test.

**Table S2** Sensitivity analysis in the average probability (column) of the old patients (rows) in multimorbidity of each class

|  | **Class 1 (%)** | **Class 2 (%)** | **Class 3 (%)** |
| --- | --- | --- | --- |
| Class 1 | 81.8 | 18.2 | 0.0 |
| Class 2 | 5.3 | 91.2 | 3.5 |
| Class 3 | 0.0 | 6.4 | 93.6 |





**Fig. S1** Sensitivity analysis in the characteristics of multimorbidity patterns
